# Preferred rehabilitation setting among stroke survivors in Nigeria and associated personal factors

**DOI:** 10.4102/ajod.v7i0.352

**Published:** 2018-07-17

**Authors:** Grace Vincent-Onabajo, Zulaiha Mohammed

**Affiliations:** 1 Department of Medical Rehabilitation (Physiotherapy), University of Maiduguri, Nigeria

## Abstract

**Background:**

Incorporating patients’ preferences in the care they receive is an important component of evidence-based practice and patient-centred care.

**Objective:**

This study assessed stroke patients’ preferences regarding rehabilitation settings.

**Methods:**

A cross-sectional design was used to examine preferences of stroke patients receiving physiotherapy at three hospitals in Northern Nigeria. Personal factors and preferred rehabilitation setting data were obtained using the Modified Rankin Scale (to assess global disability) and a researcher-developed questionnaire. Associations between preferences and personal factors were explored using bivariate statistics.

**Results:**

Sixty stroke patients whose mean age was 53.6 ± 14.8 years participated in the study. Most of the participants (38.3%) preferred an outpatient setting, 19 (31.7%) preferred rehabilitation in their homes, 14 chose inpatient rehabilitation (23.3%), while 4 (6.7%) preferred the community. Age and source of finance were significantly associated with preferences. The majority (66.7%) of those aged ≥ 65 years expressed a preference for rehabilitation in the home or community (*X*^2^ = 6.80; *p* = 0.03). Similarly, most of the participants (53.3%) who depended on family finances preferred home- or community-based rehabilitation, while most of those who depended on employment income for finances preferred an outpatient rehabilitation setting (*X*^2^ = 16.80; *p* = 0.01).

**Conclusion:**

A preference for rehabilitation in outpatient facilities predominated followed by home-based rehabilitation, and preferences varied based on age and source of finance. These variations in preferences have implications for making rehabilitation decisions.

## Introduction

Acknowledging patients’ preferences regarding the care they receive is gradually occupying a position of prominence in health care systems. With the advent of shared decision-making, patient-centred care and evidence-based practice, clinical decisions are expected to incorporate patients’ views, values and preferences (Charles, Gafni & Whelan 1997; Dawes et al. 2005; Epstein & Street 2011). Provision of individualised therapy, adherence to the ethical principle of patients’ autonomy, and facilitation of patient compliance and satisfaction with care along with improved treatment outcomes are some of the reported highlights of integrating patients’ perspectives into care (Brazier, Dixon & Ratcliffe 2009; Entwistle et al. 2010; Preference Collaborative Review Group 2008). Rehabilitation is an important and often protracted phase of care that would benefit from patient-centredness for optimal outcomes (Ozer & Kroll 2002), and this phase is particularly crucial for individuals who suffer from physically disabling diseases such as stroke.

Stroke is the third most disabling condition worldwide (Murray et al. 2012). In many developing countries such as Nigeria, the burden of stroke is assuming an alarming dimension (Johnston, Mendis & Mathers 2009; Norrving & Kissela 2013). The odds appear to be particularly stacked against stroke rehabilitation in Nigeria given the poor health care infrastructure, shortage of rehabilitation professionals, lack of country-specific stroke clinical practice guidelines and non-adherence to existing global guidelines, and the still-existing apathy towards orthodox care and embrace of traditional beliefs, perceptions and practice in stroke care in some quarters. It is therefore imperative that every approach that can boost stroke rehabilitation in the country should be considered, and this would entail incorporating stroke patients’ preferences in important aspects of rehabilitation, including the setting in which rehabilitation takes place (Magdon-Ismail et al. 2016), into care decisions. Rehabilitation setting, for instance, has been reported to affect the outcome of care (Chan et al. 2013; Couzner et al. 2013; Olaleye & Hamzat 2013).

Stroke patients’ preferences for specific rehabilitation settings have been explored in studies conducted in countries such as Jordan (AL-Oraibi et al. 2011), the United States (Gregory et al. 2010) and New Zealand (Hale et al. 2003). The home was preferred by an overwhelming majority of patients in the US and Jordan studies while no clear preference was established in the New Zealand study. It is however noteworthy that data on stroke patients’ rehabilitation setting preferences are largely unavailable in Nigeria, as in other African countries.

Stroke rehabilitation in Nigeria typically takes place during the acute in-hospital care on general medical wards or at physiotherapy gymnasia usually within the premises of the same hospitals where patients are admitted. Unlike in developed countries, there are no inpatient rehabilitation facilities in Nigeria, and nursing homes are also a rarity. Therefore, post-discharge, rehabilitation either takes place in patients’ homes or outpatient physiotherapy facilities. It is however important to state that the choices of stroke patients regarding their preferred post-discharge rehabilitation setting are often not assessed nor considered while general assumptions are usually made. The lack of inpatient rehabilitation facilities may represent one of the implications of these assumptions, especially as the impetus to establish such facilities may partly depend on consumers’ expressed preference for specific settings.

In order to gain insights into stroke patients’ preferences for rehabilitation settings that could in turn encourage incorporating such preferences into care decisions, facilitate provision of a variety of settings and possibly induce relevant health policies and strategies, this study examined the preferences of stroke patients in Nigeria regarding rehabilitation setting. A further aim was to explore the personal factors that were associated with specific preferences.

## Methods

### Study design

The study was a cross-sectional hospital-based survey.

### Participants

Consecutive consenting community-dwelling stroke patients aged 18 years and above who were able to communicate sufficiently to complete the study instrument participated in the study. The ability to communicate was verified through face-to-face interview and respective stroke patient’s medical records. All the participants were receiving post-stroke physiotherapy on outpatient basis at the time of the study.

### Study setting

The study was conducted at the physiotherapy facilities of three government-owned hospitals in Maiduguri, the capital city of Borno State in Nigeria.

### Study instruments

A researcher-developed questionnaire was used to obtain information on rehabilitation setting preference and the socio-demographic (age, gender, marital status, educational level, post-stroke employment status, source of finance and availability of social support) and clinical (side of hemiplegia, type of stroke and post-stroke duration) attributes of the participants. Information on post-stroke duration was obtained from the participants and verified using their medical records while data on the type of stroke, as diagnosed by physicians and neurologists, was solely obtained from the participants’ medical records. Another clinical attribute that was assessed was global disability, using the Modified Rankin Scale (mRS). The mRS is a valid measure of disability on a six-point scale with values ranging from 0 to 5 (Banks & Marott 2007; Van Swieten et al. 1988). A score of 0 represents no symptoms, 1 depicts no significant disability, 2 represents slight disability, 3 represents moderate disability, while 4 and 5 represent moderately severe disability and severe disability, respectively.

All the socio-demographic and clinical attributes were operationalised in this study as personal factors. To obtain data on the preferred rehabilitation setting, four options for rehabilitation setting were presented and described as follows:

Home: the physiotherapist visits and treats you at home; Community: the physiotherapist visits and treats you in a centre close to your home; Outpatient: the physiotherapist stays in the clinic and you visit to receive care and then return to your home the same day; Inpatient: you are admitted into the hospital and receive care from the physiotherapist daily.

The term ‘physiotherapist’ came up in all the descriptions of the rehabilitation settings because physiotherapists are often the sole professionals involved in stroke rehabilitation in Nigeria due to the scarcity of other rehabilitation professionals. Participants were requested to choose their preferred setting among the settings.

### Procedure

Ethical approval for the study was obtained from the Research and Ethical Committee of one of the participating institutions, while informed consent was obtained from each stroke patients who participated in the study. All data were obtained by the second author (Z.M.) through face-to-face interviews from May to July 2014.

### Data analysis

All data (age, gender, marital status, educational level, post-stroke employment status, source of finance, availability of social support, side of hemiplegia, level of global disability, type of stroke, post-stroke duration and rehabilitation setting preference) obtained were presented as descriptive statistics.

Chi-square statistics were used to examine the associations between participants’ personal factors (age, gender, marital status, educational level, post-stroke employment status, source of finance, availability of social support, side of hemiplegia, level of global disability, type of stroke and post-stroke duration) and their preferred rehabilitation setting. For the purpose of the analyses, age categories were < 65 years and ≥ 65 years; marital status categories were married and unmarried (single, divorced and widowed) while source of finance was categorised as employment income, pension, family and charity. The level of statistical significance was set at α = 0.05.

## Ethical consideration

Ethical approval for the study was obtained from the Research and Ethical Committee of the University of Maiduguri Teaching Hospital, Maiduguri, Borno State, Nigeria.

## Results

Sixty stroke patients, with a male majority (61.7%), participated in the study. The mean (standard deviation [SD]) age was 53.6 (14.8) years. Details of the socio-demographic and clinical characteristics of participants are presented in Table 1.

**TABLE 1:** Personal factors of participants (*N* = 60).

Characteristic	Value	
	*F*	%
**Socio-demographic**		
**Gender**		
Female	23	38.3
Male	37	61.7
**Marital status**		
Married	35	58.3
Unmarried	25	41.7
**Educational level**		
Nil formal	8	13.3
Below tertiary	32	53.3
Tertiary	20	33.3
**Post-stroke employment status**		
Employed	12	20.0
Unemployed	48	80.0
**Source of finance**		
Employment income	15	25.0
Pension	8	13.3
Family	30	50.0
Charity	7	11.7
**Availability of social support**		
Yes	45	75.0
No	15	25.0
**Age (years)**		
Mean ± SD	53.62 ± 14.83	
Range	18–88	
**Clinical**		
Side of hemiplegia/hemiparesis		
Right	29	48.3
Left	31	51.7
**Type of stroke**		
Ischaemic	47	78.3
Haemorrhagic	13	21.7
**Level of global disability**		
Slight disability	18	30.0
Moderate disability	26	43.3
Moderately severe disability	16	26.7
**Post-stroke duration (months)**		
Mean ± SD	16.5 ± 21.29	
Range	0.5–120	

*F*, frequency; %, percentage; SD, standard deviation.

**FIGURE 1:**
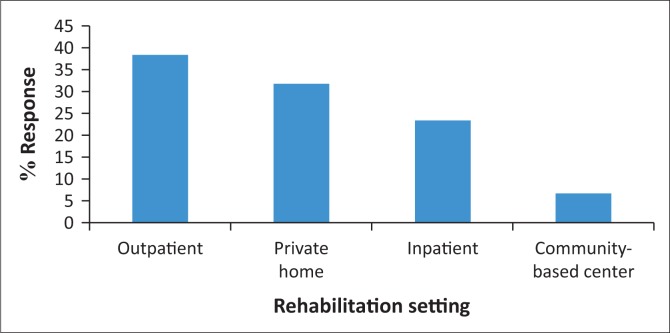
Rehabilitation setting preferences.

In terms of preferences for rehabilitation settings, 23 (38.3%) participants preferred an outpatient setting, 19 (31.7%) preferred rehabilitation in their homes, 14 chose inpatient rehabilitation (23.3%), while 4 (6.7%) preferred the community (Figure 1).

With the results of the descriptive statistics showing that only four participants preferred the community and given the fact that rehabilitation in the home and community settings both represent domiciliary rehabilitation, the ‘home’ and ‘community’ options were presented as an entity (home or community-based) for the inferential analyses. Among the personal factors explored, only age and source of finance were significantly associated with setting preferences. Table 2 shows that a majority (66.7%) of those who were ≥ 65 years expressed their preference for rehabilitation in the home or community while most (44.4%) of those who were under 65 years expressed a preference for an outpatient rehabilitation setting (*X*^2^ = 6.80; *p* = 0.03). Most (53.3%) participants who depended on family finances preferred rehabilitation in the home or community while an outpatient rehabilitation setting was preferred by most of those who depended on employment income for their finances (*X*^2^ = 16.80; *p* = 0.01).

**TABLE 2:** Personal factors associated with preferred rehabilitation setting.

Characteristic	Preferred rehabilitation setting			*χ*^2^
	Home/community *F* (%)	Outpatient *F* (%)	Inpatient *F* (%)	
**Socio-demographic**				
**Gender**				2.58
Female	9 (39.1)	11 (47.8)	3 (13.0)	
Male	14 (37.8)	12 (32.4)	11 (29.7)
**Age (years)**				6.80*
< 65	13 (28.9)	20 (44.4)	12 (26.7)	
≥ 65	10 (66.7)	3 (20.0)	2 (13.3)
**Educational level**				8.26
Nil formal	4 (50.0)	3 (37.5)	1 (12.5)	
Below tertiary	13 (40.6)	15 (46.9)	4 (12.5)
Tertiary	6 (30.0)	5 (25.0)	9 (4.5)
**Post-stroke employment status**				4.04
Employed	5 (41.7)	2 (16.7)	5 (41.7)	
Unemployed	18 (37.5)	21 (43.8)	9 (18.8)
**Marital status**				2.09
Married	12 (48.0)	9 (36.0)	4 (16.0)	
Unmarried	11 (31.4)	14 (40.0)	10 (28.6)
**Source of finance**				16.80*
Employment income	3 (20.0)	8 (53.3)	4 (26.7)	
Pension	4 (50.0)	2 (25.0)	2 (25.0)
Family	16 (53.3)	11 (36.7)	3 (10.0)
Charity	0 (0.0)	2 (28.6)	5 (71.4)
**Availability of social support**				1.17
Yes	16 (35.6)	19 (42.2)	10 (22.2)	
No	7 (46.7)	4 (26.7)	4 (26.7)
**Clinical**				
Type of stroke				0.71
Ischaemic	18 (38.3)	17 (36.2)	12 (25.5)	
Haemorrhagic	5 (38.5)	6 (46.2)	2 (15.4)
**Side of hemiplegia/hemiparesis**				1.35
Right	9 (31.0)	12 (41.4)	8 (27.6)	
Left	14 (45.2)	11 (35.5)	6 (19.4)
**Level of global disability**				3.08
Slight	4 (22.2)	9 (50.0)	5 (27.8)	
Moderate	12 (46.2)	8 (30.8)	6 (23.1)
Moderately severe	7 (43.8)	6 (37.5)	3 (18.8)

*F*, frequency; %, percentage.

* *p* < 0.05

## Discussion

Incorporating patients’ preferences into the care they receive is a cornerstone of evidence-based practice and represents an important premise of the shared decision-making model of care. An important decision regarding stroke rehabilitation is the setting in which rehabilitation takes place, and this was examined in this study.

Most participants expressed their preference for rehabilitation at outpatient facilities, closely followed by those who preferred rehabilitation at home. This finding may be considered as a reflection of the current status of stroke rehabilitation services in Nigeria. As in many developing or African countries, rehabilitation facilities are underdeveloped in the country and the only available settings for stroke rehabilitation are the private residence of the patients and outpatient facilities. Contrary to the findings of this study, however, one study conducted in the United States showed that stroke patients undergoing acute care overwhelmingly (85%) preferred rehabilitation in their homes post-acute care discharge (Gregory et al. 2010). Another study in Jordan also showed a greater preference for home-based rehabilitation among stroke patients (AL-Oraibi et al. 2011). Compared to other settings, home-based rehabilitation has been reported to allow for longer periods to carry out repetitive movement and functional training that facilitates motor relearning and ultimately, motor function recovery (Reed, Handžić & McAmis 2014).

Inpatient facility was another rehabilitation setting option in this study and it was preferred by a fifth of the participants. However, it is important to mention here that inpatient rehabilitation centres are not available in Nigeria, although rehabilitation in such centres has been reported to result in far better stroke outcomes compared to rehabilitation in other settings such as the home, outpatient or nursing facilities (Chan et al. 2013). The disturbing deficiencies in stroke rehabilitation in Nigeria are also exemplified by the fact that stroke patients are routinely managed on general medical wards while stroke units, which have been shown to produce more positive outcomes during acute stroke care, are non-existent in the country (Stroke Unit Trialists’ Collaboration 2009; Sun et al. 2013). A lot therefore needs to be done in order to standardise stroke rehabilitation in Nigeria, as in most African countries, and concerted efforts by governments, aid agencies and rehabilitation professionals would be required. Similarly, community-based rehabilitation centres are largely unavailable in Nigeria which represent a cause for concern. The lack of community-based rehabilitation centres may be responsible for the setting being the least (6.7%) preferred, especially because preference is often influenced by knowledge of the existence of, and familiarity with, specific choices.

Age was a significant personal factor that was found to be associated with preferences, and about 6 out of every 10 stroke patients who were 65 years and above in this study preferred having rehabilitation in their homes or in the community. This observation may indicate that the elderly are more comfortable at home and have psychological and emotional attachments to the family and familiar environments such as their homes. Frailty that often accompanies aging may also contribute to preference. Although the influence of frailty on rehabilitation setting preference was not explored, the study assessed level of disability but found no significant association between preference and disability level. However a previous study showed that older stroke patients were less likely to be discharged home compared to those of younger age (Nguyen et al. 2015). Similarly, some studies conducted among various groups of the elderly in developed countries showed that institutional care was preferred over home care (Gott et al. 2004; Kok, Berden & Sadiraj 2015).

Most of the participants who were financially self-sufficient preferred the outpatient setting while those who depended on others for their finances preferred rehabilitation in their private homes or the community. A study that compared stroke rehabilitation in the home versus an outpatient setting in the United States showed that stroke patients with higher incomes had more outpatient visits than those with lower income (Chan et al. 2009). With the financial burden associated with stroke, it is not surprising that finances could play an important role in patients’ preferences regarding the setting in which they receive rehabilitative care. Although information on financial cost of stroke care in Nigeria is scarce, it is important to state that the cost of domiciliary rehabilitation, particularly domiciliary physiotherapy, could be higher than the cost of outpatient care. Reasons for this difference may include the fact that pricing for domiciliary rehabilitation services is not regulated, the resource requirements in terms of transportation, time and equipment are borne by the professionals and also because such services may represent profit-making ventures for the rehabilitation professionals. The observed association between financial self-sufficiency and rehabilitation setting preference suggests that preferences may be dependent on stroke patients’ perceptions of the financial implication of rehabilitation in various settings, and such perceptions may need to be addressed by rehabilitation professionals through counselling and appropriate education.

Aside from age and source of finance, no other personal factor was found to be significantly associated with preference for rehabilitation setting in this study. However it is important to note that some previous studies did not dwell on the effect of personal factors on rehabilitation setting preference (AL-Oraibi et al. 2011; Hale et al. 2003). Rather, they explored the association between setting preference and factors such as cost, transportation and waiting time, with results showing that these factors tipped some of the stroke survivors in favour of home-based rehabilitation (AL-Oraibi et al. 2011; Hale et al. 2003). Reports also show that preference for outpatient setting was linked to satisfaction with the availability of the array of equipments, opportunity to socialise and respite for carers (Hale et al. 2003; Thomas & Parry 1996). The fact that opportunity to socialise was found to be so important to stroke survivors that it influenced their preference for outpatient rehabilitation setting as reported in the studies cited comes as no surprise. For instance, previous studies have shown that social support has positive and significant impact on important variables such as participation (Vincent-Onabajo et al. 2016a) and quality of life (Vincent-Onabajo et al., 2016b) of stroke survivors. However, the present study found no significant association between availability of social support (or otherwise) and rehabilitation setting preference. Future studies may therefore be required to assess the impact that specific factors such as socialisation opportunity and the desire for support for caregivers (in the form of caregivers’ respite) have on rehabilitation setting preference instead of the effect of global social support, as was done in this study. Furthermore, future studies on other factors that could influence preferences such as the patients’ psychological status, frequency of stroke (whether first-ever or recurrent stroke) and views about efficacy and quality of care are required in our setting. Prospective studies with larger samples are also needed to examine the trend of preferences across the stroke continuum.

## Limitations of the study

The limitations of the present study include the small size of the sample, which precluded data treatment using more vigorous statistics such as multiple regression analyses to identify independent determinants of preferences. Similarly, the small sample size could have been responsible for the very low number of participants that registered their preference for rehabilitation in the community. This necessitated grouping the home and community settings as an entity for the purpose of the inferential analyses, although it is important to mention that the two settings represent a domiciliary rehabilitation setting. The hospital-based design of the study may also limit the generalisability of the findings and future community-based studies would therefore be beneficial. In the same vein, the fact that the participants were all undergoing outpatient physiotherapy at the time of the study could have biased the results of the study and resulted in some participants’ preference for the familiar. Although the impact of patients’ preference on the outcome of care was outside the scope of this study, it is important to note that patients’ preferences may not always be realistic, feasible or in their best interest and this should be borne in mind when interpreting the findings. Also, given the debilitating effect of stroke on several body functions including mental functions, obtaining information on stroke patients’ preferred rehabilitation setting may not always be possible or feasible.

## Conclusion

The distribution of the preferences for rehabilitation setting in this study coupled with the variations in preferences based on the stroke patients’ personal factors highlight the variability of individual patient preferences and supports the need for patient-centred stroke rehabilitation procedures that assess patients’ preferences and take them into consideration when decisions about rehabilitation settings are made.

### Practical implications

Assessment of stroke patients’ preferences for rehabilitation practice should constitute routine practice given the variations in preferred setting among different categories of patients.
